# Anticholinesterase Activities of Different Solvent Extracts of Brewer’s Spent Grain

**DOI:** 10.3390/foods10050930

**Published:** 2021-04-23

**Authors:** Rares I. Birsan, Peter Wilde, Keith W. Waldron, Dilip K. Rai

**Affiliations:** 1 Department of Food BioSciences, Teagasc Food Research Centre Ashtown, D15KN3K Dublin, Ireland; Rares.Birsan@teagasc.ie; 2 Food Innovation and Health Programme, Quadram Institute Bioscience, Norwich Research Park, Colney NR4 7UQ, UK; Pete.Wilde@quadram.ac.uk; 3 Anglia Science Writing Ltd., Wramplingham, Norfolk NR18 0RU, UK; keithwwaldron@outlook.com

**Keywords:** brewer’s spent grain, polyphenols, acetylcholinesterase, butyrylcholinesterase.

## Abstract

Cholinesterases, involved in acetylcholine catabolism in the central and peripheral nervous system, have been strongly linked with neurodegenerative diseases. Current therapeutic approaches using synthetic drugs present several side effects. Hence, there is an increasing research interest in naturally-occurring dietary polyphenols, which are also considered efficacious. Food processing by-products such as brewer’s spent grain (BSG) would be a potential bio-source of polyphenols. In this study, polyphenol-rich BSG extracts using 60% acetone and 0.75% NaOH solutions were generated, which were further subjected to liquid–liquid partitioning using various organic solvents. The water-partitioned fractions of the saponified extracts had the highest total polyphenol content (6.2 ± 2.8 mgGAE/g dw) as determined by Folin–Ciocalteu reagent, while the LC-MS/MS showed ethyl acetate fraction with the highest phenolics (2.9 ± 0.3 mg/g BSG dw). The best inhibitions of acetyl- (37.9 ± 2.9%) and butyryl- (53.6 ± 7.7%) cholinesterases were shown by the diethyl ether fraction of the saponified extract. This fraction contained the highest sum of quantified phenolics (99 ± 21.2 µg/mg of extract), and with significant (*p* < 0.01) inhibitory contribution of decarboxylated-diferulic acid. Amongst the standards, caffeic acid presented the highest inhibition for both cholinesterases, 25.5 ± 0.2% for acetyl- and 52.3 ± 0.8% for butyryl-cholinesterase, respectively, whilst the blends insignificantly inhibited both cholinesterases. The results showed that polyphenol-rich BSG fractions have potentials as natural anti-cholinesterase agents.

## 1. Introduction

Evidence in the current literature suggests a strong link to the protective effects of dietary polyphenols towards the prevention of so called “diseases of civilization”, i.e., chronic non-communicable diseases, and protective effects justified via the “biochemical scavenger theory” [1,2]. Not only being the most abundant antioxidants present in human diet, researchers, food companies as well as consumers, consider dietary polyphenols to be one of the core groups of dietary preventive agents [3].

Alzheimer’s disease (AD), the most common type of dementia, is a progressive neurodegenerative disease that is commonly characterized by the presence of amyloid-β deposits, τ-protein aggregation, low level of acetylcholine and oxidative stress [4]. More than 115 million people worldwide are estimated to be affected by this disease by 2050 with most of individuals aged over 65 years [4]. Even though the AD pathogenesis has not been fully understood, the main mechanistic theory proposed is the “cholinergic hypothesis” [5]. Choline is an important quaternary amine responsible for the structural integrity and signaling functions of cell membranes, which directly affects the cholinergic neurotransmission [6]. Acetylcholine and butyrylcholine are important metabolites of choline; acetylcholine is the main neurotransmitter at autonomic preganglionic nerve terminals and mostly prevalent in cholinergic synapses of the central and peripheral nervous system [7,8]. A decrease of acetylcholine levels in the cholinergic synapses in the brain regions seems to be a critical element in the development of AD. Cholinesterases, i.e., acetylcholinesterase (AChE) and butyrylcholinesterase (BChE), are enzymes that hydrolyze acetylcholine and butyrylcholine, respectively, and their inhibition is a current therapeutic target [4]. Synthetic drugs prescribed to inhibit cholinesterase’s activity have known to have side effects including nausea, vomiting, headache, etc. However, some plant-derived alkaloids such as galantamine, tacrine and physostigmine are also used and have shown symptomatic improvement in AD [5].

Research studies in recent years have led to belief that the polyphenols, as natural antioxidants, play a role in prevention and management of numerous degenerative diseases by reducing the oxidative stresses generated by free radicals and oxidants [9,10]. Several studies have shown a solid association between foods rich in polyphenols and the reduction of oxidative stress and amyloid accumulation in AD patients [11,12,13]. Specific phenolic acids such as ferulic acid, caffeic acid, *p*-coumaric acid, and 4-hydroxybenzoic acid among others have received considerable attention as anti-inflammatory agents in the pathogenesis of chronic diseases including cancer and cardiovascular diseases [14,15,16,17,18]. Coincidently, the aforementioned phenolic acids are present in high abundance in brewer’s spent grains (BSG) [19,20,21]. The main hurdle in BSG is that these phenolic acids are generally bound to other cell wall components requiring hydrolysis by chemical or enzymatic methods for their extraction [22,23,24]. In essence, BSG is a lignocellulosic material comprising approximately 80% cell wall material and the remaining 20% consists mainly of proteins [25]. Saponification with sodium hydroxide (NaOH) at different concentrations is an efficient method for liberation of ester and ether-linked phenolics from xylan, hemicelluloses and lignin components [26]. Such extraction processes are necessary to generate extracts with high polyphenol yield. Solid–liquid extraction and subsequent liquid–liquid extraction are the most frequently used procedures for this purpose due to ease of use, efficiency and broad applicability [27].

Further separation and enrichment of these hydrolyzed constituents depend greatly on the suitability of the extraction process, phase separation of the initial solvents besides other extraction parameters (temperature, time, pH, etc.) However, a great influence on the recovery of the constituent compounds is the choice of solvent used. Laws of similarity and miscibility suggest that it is more likely for a solute to dissolve in a solvent close to its polarity. Phenolic acids are categorized as hydrophilic or polar compounds and have been successfully fractionated and purified from complex mixtures by using mid-polar range solvents, i.e., ethyl acetate. Although previous research studies have shown several extraction techniques for the recovery of bound polyphenols from cereal and cereal-based products, a limited information exists for the BSG polyphenols as the majority have focused only on using organic solvents or water to extract free phenolics [22,28,29,30,31].

With a hypothesis that phenolic rich extracts from BSG could be efficient inhibitors against AChE and BChE activities, this study aimed: (1) To extract free phenolics using 60% acetone, and bound phenolics using saponification with 0.75% NaOH from BSG; (2) to assess the efficiency of four different organic solvents (hexane, diethyl ether, ethyl acetate, butanol) in recovery of phenolic compounds from the two extracts; (3) to determine the total phenolic content and quantify the major phenolic compounds in the extracts and organic solvent fractions; and (4) to determine the anti-cholinesterase activities of the extracts and fractions along with the individual and mixtures of quantified phenolic compounds detected.

## 2. Materials and Methods

### 2.1. Materials and Chemicals

BSG was provided by Diageo Dublin, Ireland, which was directly transported to the research centre within 30 min, oven-dried (Binder E28 oven, 72 h, 60 °C), milled (<1 mm), vacuum packed and stored at −28 °C until required.

The organic solvents, methanol, ethanol (EtOH), acetone (Ace), *n*-hexane (Hex), diethyl ether (DE), ethyl acetate (EtOAc), *n*-butanol (BuOH), acetonitrile, formic acid, hydrochloric acid (HCl), and sodium hydroxide (NaOH) were purchased from Merck (formerly Sigma Aldrich, Arklow, Co. Wicklow, Ireland). Polyphenol standards, *p*-coumaric acid (*p*-CA), ferulic acid (FA), caffeic acid (CafA), protocatechuic acid (ProA), 4-hydroxybenzoic acid (4-HBA) and +(-)catechin (Cat), chemicals (sodium carbonate, sodium chloride, magnesium chloride, tris hydrochloride, tris base), reagents (Folin Ciocalteu, Ellman’s or 5,5′-Dithiobis(2-nitrobenzoic acid)( DTNB), inhibitor standard (galantamine hydrobromide from *Lycoris* sp.), proteins (bovine serum albumin), substrates (acetylthiocholine iodide, s-butyryl thiocholine iodide) and enzymes (acetylcholinesterase from electric eel, butyrylcholinesterase from equine serum) necessary to determine in vitro the total phenolic content and cholinesterase inhibitory activities were purchased from Merck (formerly Sigma Aldrich, Arklow, Co. Wicklow, Ireland). All chemicals used were of analytical grade and all solutions were prepared with milli-Q water.

### 2.2. Solid–Liquid Extraction of Free and Bound Phenolics

Extraction of free- (FP) and bound-phenolic (BP) compounds from BSG was done by maceration in combination with 60% acetone and 0.75% NaOH solution, respectively, following the methods of [32,33] with small changes. Briefly, 7 g milled BSG was mixed with 140 mL of solvent (1:20 *w/v*) in a sealed amber glass bottle and kept in a water-bath at 60 °C (free phenolic extraction) and at 80 °C (bound phenolic extraction) for 30 min with constant stirring.

**Figure 1:**
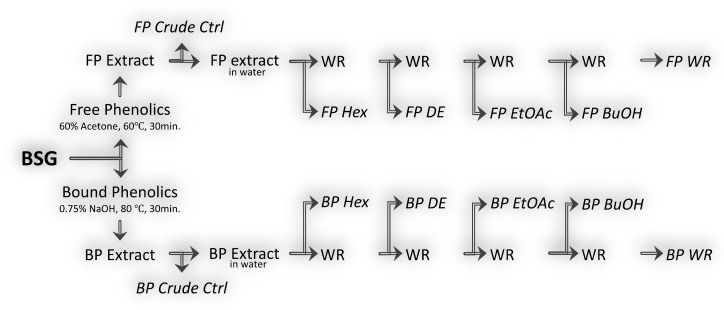
Extraction process of free and bound phenolic compounds from BSG followed by their partitioning using different organic solvents. FP—free phenolics, BP—bound phenolics, WR—water residue, Hex—hexane, DE—diethyl ether, EtOAc—ethyl acetate, BuOH—butanol, Ctrl—control.

After the treatment time, all the extracts were left to cool at room temperature followed by centrifugation at 9484 *g* for 10 min (Sigma 2–16KL, Osterode am Harz, Germany). The supernatants were pooled, syringe filtered through 0.45 μm PTFE filters for FP extracts, whereas the BP extracts were neutralized to pH 6.5 and paper-filtered under vacuum. The FP and BP extracts were stored at −28 °C until required. The extraction of FP and BP from BSG was carried out in quadruplicates, from which three were used further for liquid–liquid partitioning (fractions) and one as control (crude) as illustrated in (Figure 1).

### 2.3. Liquid–Liquid Partitioning of Free and Bound Phenolic Extracts

The fractionation of the FP and BP extracts with different solvents of polarity was adapted from Tu et al., 2013 [34] with some modifications. Both the FP and BP extracts were fractionated by using solvents with increasing polarity (empirical parameters of normalized solvent polarity shown in brackets after each solvent) as follows: *n*-hexane (0.009), diethyl-ether (0.117), ethyl acetate (0.228), *n*-butanol (0.586) saturated by water (1.0), and the residual water as the remaining fraction [35]. The FP extract was concentrated under vacuum (Rotavapor R-100, Buchi, Switzerland) to evaporate the acetone and the remaining water part (~50 mL) was used for liquid–liquid partitioning (Figure 1). The recovered volumes of aqueous FP and BP extracts were sequentially pooled three times with each organic solvent in equal volumes of water. The organic layer was recovered and concentrated under vacuum (38 °C), whereas the remaining residual water fraction (WR) was freeze dried. The recovered dried material was reconstituted in a minimal volume of ethanol (98%, *v/v*) and further diluted with double distilled water to a final concentration of 20 mg/mL, which served as stock solution. The final fractions were syringe filtered as above and stored in a freezer at −28 °C until further use.

### 2.4. Determination of Polyphenolic Content

Total phenolic content was estimated by Folin–Ciocalteu and quantification of BSG polyphenols in the FP and BP extracts and fractions was performed by LC-MS/MS as described previously [21].

#### 2.4.1. Total Phenolic Content (TPC) by Folin–Ciocalteu (FC)

Briefly, in a 1.5 mL Eppendorf tube, 100 µL of extract, 100 µL of ethanol (for Hex, DE, EtOAc and BuOH fractions) or milli-Q water (for WR fraction), 100 µL of FC reagent and 700 µL of 20% sodium carbonate solution were added, vortexed, and incubated for 20 min. in darkness at room temperature. The reaction mixture was then centrifuged (13,000 rpm, 3min) from which 200 µL was transferred onto 96-well micro plate and measured for absorbance at 735 nm using a spectrophotometer (FLUOstar Omega, BMG Labtech, Germany). Gallic acid was used as standard at various concentrations (10–300 μg/mL in 50% ethanol) to prepare a calibration curve (y = 0.0095x − 0.0124, *r^2^* = 0.998). The results are expressed in milligrams of gallic acid equivalent per gram (mg GAE/g) of BSG extract or fraction.

#### 2.4.2. Individual Polyphenol Quantification by UPLC-MS/MS

Ultra-high performance liquid chromatography coupled to tandem quadrupole (UPLC-TQD) mass spectrometer (Waters Corp., Milford, MA, USA) was used to quantify the most abundant and identified polyphenols as described previously [21].

For the quantification of polyphenols, appropriate dilutions (0.098 to 50 ppm) of each standard (FA, *p*-CA, Cat, CafA, 4-HBA, ProA) were prepared to obtain a standard calibration curve. Targetlynx^TM^ (Waters Corp., Milford, MA, USA) software was used to quantify the compounds in the various extracts. The ferulic acid dimers and trimers were quantified using the standard curve from FA (y = 1064.59x + 12.24, *r^2^* = 0.99).

### 2.5. Preparation of Polyphenol Blends

In order to associate the anti-cholinesterase activity of the BSG fractions to their polyphenol content, blends that mimic the polyphenol content in the BSG fractions were prepared and tested separately. Thus, six polyphenols were used in combination to prepare three blends that mimic their abundance in BSG fractions. The blends were prepared at a specific polyphenol ratio as calculated by their UPLC-MS/MS quantification to a final concentration of 1000 μg/mL. For this purpose, the fractions that presented the highest content of quantified polyphenols were selected, namely Blend FP1 EtOAc, Blend BP1 DE, Blend BP3 EtOAc; the number 1 or 3 following Blend FP or BP represents the replicate fraction number that was used to prepare the blend. The specific polyphenols combinations are presented in Supplementary Table (Table S1).

### 2.6. Anti-Cholinesterase Assays 

The inhibitory potential of BSG extracts, fractions, blends and individual polyphenol towards anti-AChE and anti-BChE activities was determined in vitro by Ellman’s colorimetric method [36] and adapted to cuvettes following the procedure of Faraone et al., 2019 [37]. The ethanol content of the samples was lower than 5% (*v/v*) in the final assay mixture and the interference on the enzyme activity was subtracted from the final % inhibition calculations.

For the AChE assay, 75 μL of sample (1 mg/mL extract in final assay mixture), 150μL of 50 mM Tris HCl buffer (pH 8 with 0.1% bovine serum albumin), 375μL of 3 mM DTNB reagent and 75μL of 15 mM acetylcholine iodide substrate were added in a cuvette and pipette mixed. The reaction was initiated by adding 75μL of 0.18U/mL AChE enzyme solution and pipette mixed. A blank solution containing 75 μL of 50 mM Tris HCl buffer instead of enzyme solution for each individual sample was used to zero the spectrophotometer prior to reaction initiation. Similar steps were followed for BChE assay, where the substrate (75 µL of 15 mM S-butyrylthiocholine chloride) and the enzyme (0.1 U/mL of BChE) were used instead. The change in absorbance at 405 nm was recorded for every minute up to 5 min using Shimadzu PharmaSpec UV-1700 spectrophotometer (Shimadzu Scientific Instruments, Columbia, MD, USA). Galantamine, a cholinesterase inhibitor and a commonly prescribed drug for treating AD, was used at different concentrations (1.56 to 50 μg/mL for AChE and BChE in 50% ethanol) as positive control, and the required concentration to inhibit the activity of AChE and BChE by 50 percent (IC_50_) was calculated by nonlinear regression analysis. The rate of reaction over time (slope) was calculated for each recorded sample in duplicate against negative control (NC, 50 mM Tris HCl buffer instead of sample/inhibitor), and the final results were expressed as percentage of inhibition:

%Inhibition = (1−AbsSlope(Sample)/AbsSlope(NC)) × 100.(1)

### 2.7. Statistical Analysis

Results are expressed as means of the triplicates ± standard deviation (SD). The datasets were evaluated for normality and homogeneity of variance by Shapiro–Wilk and Levene’s test. Normally distributed data sets were evaluated using one-way ANOVA and Tukey’s post hoc tests, whereas non-normal distribution by nonparametric Kruskal–Wallis and Dunn’s post hoc test (*p* < 0.05). Welch analysis followed by Games-Howell post hoc test were performed when Levene’s test (homogeneity of variance) was significant (*p* < 0.05). The correlation coefficients between the measured variables were calculated using Pearson correlation (*p* < 0.05), and the relation was assessed by regression model (dependent variables: AChE and BChE, independent variables: quantified phenolic compounds and their quantification methods, i.e., TPC by FC and SQP by UPLC-MS/MS). The statistical analytical steps were followed as proposed by Granato et al., 2014 [38]. Principal component analysis (PCA) was carried out with the standardized data sets to disclose any association between the quantified phenolic compounds in the extracts and the enzymatic assays. Statistical analysis, Pearson’s correlation and linear regression were carried out using SPSS v.25 (IBM corp.), while PCA using Minitab v.17 (Minitab, Inc., Coventry, UK).

## 3. Results and Discussion

### 3.1. Extraction Yield

The extraction yields were measured first for the crude extracts with and without saponification, and then for the different solvent fractions employed in liquid–liquid partitioning (Table 1). Extraction yield defined as “Total” yield in (Table 1) was determined by summing yield of each of the various liquid–liquid fractions. As exemplified by the 60% acetone extract, the yield of total fractions (80.2 ± 3.4 mg/g BSG dw) was lower than that of the crude extract (94.9 ± 9.2 mg/g BSG dw) indicating the occurrence of losses during the liquid–liquid partitioning, such as emulsion formation, through the filtration process as well the variation of the sample material, particle size, solubility of the immiscible solvent, when extractions were done in replicates [39]. The extraction yield of total fraction following saponification (0.75% NaOH) showed a 5-fold higher yield (424.2 mg/g BSG dw) than the unsaponified (60% acetone, 80.2 mg/g BSG dw). More than 80% of the saponified material was recovered in the WR fraction followed by 9% in EtOAc and 6% in BuOH fractions. On the other hand, for the unsaponified (60% acetone) extract, recovery in the Hex, DE and WR fractions were in similar range amounting to 32%, 28%, and 25%, respectively of the total recovered material.

The results presented in (Table 1) were generated by solid–liquid and liquid–liquid extractions, followed by paper filtration and concentrated under vacuum or freeze-dried. As the extractions were carried out in triplicate, the steps of washing the solid extraction residue (crude extracts) and separation of the immiscible solvents (Hex, DE, EtOAc, BuOH, and water) had influenced the extraction yield levels. Other parameters that may influence the variations in the extraction yield include extraction time, temperature, solvent-to-sample ratio, the number of extractions of the samples and the solvent type [40]. BSG is comprised of about 80% lignocellulosic material mainly consisting of polymers, such as cellulose, hemicellulose and lignin, originating from the cell wall material, whereas the remaining 20% comprises mainly proteins [25,41]. Saponification with NaOH facilitates the delignification of BSG and degradation of other constituents including hemicellulose and proteins [42,43], and thereby solubilizing between 23% and 60% of the current total BSG constituents (Table 1, BP total extraction yield).

Solvent extraction is a suitable method for pooling free base forms of non-saccharide components such as phenolics and other components [22], where a recovery of up to 9% of total BSG constituents was observed in this study (Table 1, FP total extraction yield). Several authors have also showed alkaline treatment is more effective than organo-solvent method in populating high extraction yields [22,44,45]. Beside delignification, dilute alkali solutions are predominantly used to hydrolyze hemicelluloses to mono-sugars/oligomers or proteins into its constituent amino acids and peptides, which can be recovered in the water phase [44]. The presence of such non-polyphenolic molecules could explain for the high variation in the standard deviation and the data is being skewed by the water fraction as it contains all the precipitates of polysaccharides, proteins, etc. Values on the extraction yield in this study are similar to those reported by other authors [46,47]. It is essential to obtain a consistent extraction yield so that the extraction process is economically feasible [48].

### 3.2. Total Polyphenol Content

Two different methods had been used to determine the total polyphenols, i.e., colorimetric method for total phenolic content (TPC) using FC reagent, and sum of quantified polyphenols (SQP) by UPLC-MS/MS method (Table 1). The results revealed a considerable variability in the TPC and SQP values, where TPCs were always higher than SQP, among the BSG extracts and various solvent fractions. Interestingly the total bound phenolics (BP) presented almost 20 times higher TPC than free phenolics (FP), which was further supported by the SQP values. Overall, the highest TPCs were observed in the WR and EtOAc fractions of the alkali-hydrolyzed extracts with 6.2 ± 2.8 and 3.5 ± 0.5 mg GAE/g BSG dw, respectively. Amongst the FP fractions, the highest TPC was in the DE fraction (0.23 ± 0.09 mg GAE/g BSG dw) and the TPC values below 0.12 ± 0.07 mg GAE/g BSG dw were observed for the other solvent fractions. On contrary, the highest SQP was found in the EtOAc and lesser in DE fractions with 2.9 ± 0.3 and 0.8 ± 0.05 mg/g BSG dw, respectively. The FP fractions presented a very low SQP (<0.04 mg/g BSG dw) or at not-detectable levels in the Hex and WR fractions. In the authors’ previous work [21], the TPC values of BSG EtOAc fraction generated by maceration were about 33% higher than the values reported in this study even though the substrate supplier was the same but from a different malted batch. The different sample-type would factor in TPC variation alongside its background such as barley variety, harvesting time, brewing process, extraction process, etc. [22]. A significant (*p* < 0.01) correlation has been observed between the extraction yield and TPC (*r* = 0.896) using both FP and BP methods of extraction with their independent fractions.

There was a high variation (~33 fold) between the TPC values reported in the literature by numerous authors either in BSG extracts or fractions generated using alkali hydrolysis or organic solvents; the TPC values varying between 0.6 to 10 mg GAE/g dw when using organic solvents and up to 20 mg GAE/g dw when using alkali hydrolysis [22,49]. Results from this study fall within this range (Table 1). EtOAc is a commonly used organic solvent as an extractant, and is also generally recognized as safe for food application by US-FDA and EFSA to recover phenolic compounds. Meneses et al., 2013 extracted antioxidant phenolic acid from BSG using different organic solvents and/or in combination with water and showed that all the extracts presented TPC along with lower amounts of proteins and reducing sugars [32]. Meneses et al., 2013 also showed that the antioxidant activity of the extracts correlated with the total phenols and flavonoids, and acknowledged that some antioxidant activity contribution came from compounds that were not identified. Similarly, Kähkönen et al., 1999 reported that TPC can be influenced by specific compounds present in mixtures, and therefore can result in a false prediction of the antioxidant activity based only on TPC values [50].

LC-MS/MS quantification of individual phenolics, expressed as Sum of Quantified Phenolics (SQP), in the bounds phenolic (BP) extracts showed that DE and EtOAc extracts accounted for approximately 21% and 76% of the Total SQP, respectively, which corresponded to 6% and 30% of the total TPC values, respectively. In addition, DE and EtOAc fractions of BP extracts showed similar TPC and SQP trends suggesting both organic solvents were able to efficiently extract phenolic compounds from aqueous solutions. For the BP (Hex, BuOH, and WR) fractions, the SQP values were extremely low, which were also noted low in the corresponding FP fractions for both the SQP and TPC values (Table 1).

The overestimation of the spectrophotometric over chromatographic method on total polyphenol content is a well-known phenomenon as the former crudely estimates end- products by both phenolic and non-phenolic compounds [21]. One must use organic solvents such as DE or EtOAc or in combination to pool phenolic compounds from aqueous extracts, which further can be more accurately determined by spectrophotometry (TPC) and liquid chromatography-tandem mass spectrometry (LC-MS/MS).

### 3.3. UPLC-MS/MS Quantification of BSG Free and Bound Polyphenols

As previously described [21], 14 different polyphenols were tentatively identified in the EtOAc fraction of the saponified BSG extract, of which 8 were confirmed using commercially available standards in the UPLC-MS/MS method. In the current work, a total of 9 different polyphenols were quantified, five phenolic acids (ferulic acid, *p*-coumaric acid, caffeic acid, 4-hydroxybenzoic acid, and protocatechuic acid) and a flavonoid (catechin), along with two ferulic acid oligomers, (decarboxylated diferulic acid (DeCa-DiFA), diferulic acid (DiFA), and a trimer, triferulic acid (TriFA)), as ferulic acid equivalents (Table 2).

The most predominant phenolic acid, i.e., ferulic acid, was measured in the BP fractions, specifically in the DE and EtOAc fractions constituting in excess of 42% and 48%, respectively of the total polyphenols. The next abundant phenolic acid was *p*-coumaric acid with 26% and 19% in the DE and EtOAc fractions, respectively. DeCa-DiFA was the most abundant ferulic acid dimer in the BP DE fraction (31% of the total polyphenols), whereas it was present in traces in the rest of fractions. DiFA and TriFA were found in similar quantities in the BP EtOAc fraction constituting approximately 15% of the total polyphenols, but very low or not detected in the other BP solvent fractions. Catechin was the most abundant polyphenol in FP fractions, representing more than 72% and 61% of the total polyphenols in the EtOAc and DE fractions, respectively. DE and EtOAc showed to be the best solvents to recover phenolic (FP and BP) compounds from BSG. Both DE and EtOAc, due to their ability to form biphasic with water, where the extraction of mid-polar to non-polar BSG polyphenols is facilitated. Almost 98% of the total phenolic compounds in BSG, as quantified by the UPLC-MS/MS, were present in bound form, whereas the rest 2% were in the free form. These results are in similar range with previous published papers [21,45].

Stalikas 2007 comprehensive review on general polyphenols and flavonoids noted several authors had successfully used DE and EtOAc to extract phenolic compounds from aqueous solutions [27]. de Simon et al., 1990 showed there was not a very large difference in the extraction rate of EtOAc compared to DE [51]. EtOAc presented a greater extraction rate for acids and aldehydes of low and high molecular mass, such as catechin (dimers, trimers of catechins), hydroxycinnamic esters, whereas DE showed a superior reproducibility for the extraction of aldehydes and phenolic acids, i.e., 4-HBA aldehyde, *p*-CA [51]. It is for this reason some authors used a ratio of 1:1 (EtOAc:DE) to fractionate phenolic compounds from aqueous solutions [52]. Meneses et al., 2013 showed that hexane was able to extract flavonoids from BSG in low amounts [32], although hexane is mainly used to extract highly nonpolar compounds such as waxes, oils, sterols or for delipidation purposes [22]. Socaci et al., 2018 had shown hexane to be a possible selective solvent for other classes of bioactive called terpenoids and aroma compounds [29]. *n*-butanol and water are usually used to extract polar compounds such as phenolic glucosides, peptides and sugars [53].

BSG is a good source of phenolic acids, such as hydroxycinnamic acids (FA, *p*-CA) and hydroxybenzoic acids (4-HBA), and smaller amounts of flavan-3-ols such as catechin [32,33,41,54]. Among the phenolic compounds present in BSG, hydroxycinnamic acids, namely FA and *p*-CA, are the most abundant phenolics as observed in this study and by several other authors [54,55]. The highest yield of 1.31 ± 0.04% of BSG dw has been reported for FA, and a 10 fold lower values were reported by the same authors in their later published article following saponification with NaOH [33,56]. The latter FA values are in accordance with our presented results and with most of the other authors [57,58]. *p*-CA has been reported in levels of 2 to 3 fold lower than FA [56], in close range to FA [41,59] and sometimes only traces were observed [60]. Catechin, the most common flavonoid reported in BSG, can be extracted by using organic solvents without the need of saponification, and its content is reported to be below 10 mg/g BSG dw [49,61], which are in similar levels in this study. The remaining phenolic acids (caffeic acid, 4-hydroxybenzoic acid and protocatechuic acid) were mostly reported in literature at very low levels compared to FA or *p*-CA [54,62]. It can be clearly seen (Table 2) that DE and EtOAc were the best solvents to recover the above-mentioned variety of polyphenols, either using extraction with 60% acetone or saponification with NaOH, whereas only traces or low amounts could be found in Hex, BuOH, and WR, respectively. Several authors observed the loss of phenolic acids during harsh alkali hydrolysis (2–4M NaOH solution), but not beyond 10% of the initial values of ferulic and *p*-coumaric acids. However, a stronger alkali condition led to 67% and 36.5% losses of caffeic and sinapic acids, respectively [52,63]. Beside the above quantified polyphenols, procyanidin B, and chlorogenic acid have been detected in FP EtOAc and BuOH fractions, and sinapic acid in BP and EtOAc fractions. Martín-Garcia et al., 2019 extracted high yield of proanthocyanidin compounds (catechins, procyanidins) from BSG using aqueous acetone, where up to 0.1% BSG dw proanthocyanidins was extracted [61]. Therefore, depending on the bioactive compounds of interest, different optimized extractions and a variety of organic solvents are required to obtain high extraction yields of the targeted compounds.

### 3.4. Anti-AChE and -BChE Activities

The inhibitory activities of BSG free and bound phenolic extracts along with their various solvent fractions on AChE and BChE were evaluated in-vitro. The inhibition results are summarized in (Table 3) along with the TPC and SQP (µg GAE/mg and µg/mg of BSG extract or fraction) contents of the tested samples with their corresponding inhibitory potential (in %) of AChE and BChE activities. Samples were tested at a concentration of 1 mg/mL BSG extract in the final assay mixture, unless otherwise stated. It is worth to mention that the sum of quantified polyphenols in FP1 EtOAc, BP1 DE and BP3 EtOAc fractions (fractions chosen for blend preparation) represented 2.08, 123.4, and 96.5 μg/mg of BSG fraction respectively, whereas by difference to 1000 μg comprises of other unidentified compounds. All the tested samples exhibited some degree of inhibition on both AChE and BChE with the overall highest inhibitions coming from the BP fractions. FP WR fraction was the only fraction that did not present BChE inhibition. BP DE fractions showed the highest and similar TPC and SQP values with BP EtOAc fraction, while showing 4 and 2-fold higher inhibitions for AChE and BChE activities, respectively. In contrast, FP BuOH fraction showed significantly lower TPC and SQP compared to BP DE, whilst presenting similar inhibitory activities for both AChE and BChE. BP DE fraction presented similar levels of individually quantified phenolic acids with BP EtOAc fraction, with the exception of ferulic acid dimers. DeCa-DiFA was the most abundant polyphenol in BP DE fractions, whereas DiFA and TriFA were present only in BP EtOAc (Table 2). The presence of DeCa-DiFA only in BP DE fraction may be responsible for the higher inhibitory potential of this fraction towards AChE and BChE activity. This is supported by a significant correlation observed between DeCa-DiFA and anti-AChE/BChE activities (Table S3). Pure FA standard was tested individually for anti-AChE and BChE activity (Table 4), but neither the dimers nor trimers of FA could be tested individually as they are not commercially available. Adelakun et al., 2012 showed that ferulic acid dimers have higher antioxidant capacity than the ferulic acid [64]. The FA dimers have four free hydroxyl groups compared to FA (two groups) which could contribute to antioxidant efficacy [65]. Even though multiple hydroxyl groups in the phenolic compounds are thought to boost the inhibitory action of AChE through strong ionic binding capacity, unfortunately not all follow the same mode of action due to conformational variation [66].

Even though various structural isomers of ferulic acid dimers and trimers obtained from several sources had been described in the literature, there is a lack of information on their antioxidant capacity or as potential enzyme inhibitors, especially of DeCa-DiFA [67]. Jia et al., 2018 synthetized and evaluated several diferulic acids for antioxidant activity and showed DeCa-DiFA as the best antioxidant among other ferulate dimers examined. Unfortunately, no conclusive explanation for the higher inhibitory capacity of DeCa-DiFA, and rather a mix of associated structural characteristics and physiochemical properties of the compounds [68]. Furthermore, decarboxylation of ferulic acid changes the antioxidant capacity of ferulic acid, and the product formed (4-vinylguaiacol) is a potent antioxidant comparable to α-tocopherol [69]. It has been demonstrated that in homogenous polar mediums, ferulic acid presents a greater antioxidant capacity compared to its vinyl derivate 4-vinylguaiacol, whereas in emulsion systems the antioxidant capacity of 4-vinylguaiacol is much greater [70]. Further investigations are needed as to understand how DeCa-DiFA present a higher inhibitory capacity against both AChE and BChE activities compared to other related compounds.

Ouattara et al., 2013 showed that inhibitions of AChE activity decreased in the order BuOH > EtOAc fractions of *Nelsonia canescens*, even though the EtOAc fraction presented considerable higher polyphenol content (hydroxycinnamic acids) as well as antioxidant activity [71]. Due to low recovery in one of the FP BuOH replicate fractions, a solution of 0.1 mg/mL fraction was tested that showed an AChE inhibition of 11.1 ± 0.95% and 12.1 ± 1.25% for BChE inhibition. Another fraction, i.e., BP DE was tested at 0.5 mg/mL and showed an inhibition of 9.5 ± 2.05% towards AChE and 38.95 ± 3.94% for BChE inhibitions. This fraction presented the highest SQP content and was tested at a 2-fold dilution to check if the % inhibition is concentration dependent.

Several authors have showed that extracts with considerable higher polyphenols content and antioxidant activity (EtOAc extracts), obtained from different plant sources did not exhibit higher inhibitory potential for AChE and BChE activities [71,72]. It may be that the contribution of other unidentified bioactive compounds that constitute up to 99% and 90% of FP BuOH and BP DE fractions, respectively, account for the inhibition of AChE and BChE activities. Therefore, further separation of these fractions is required to assign their individual involvement in inhibition of AChE and BChE activities, which is beyond the scope of this study. In an earlier study on extracts rich in hydroxycinnamic acids from 26 medicinal plants of the *Lamiaceae* family were tested at 0.25, 0.5, and 1 mg/mL against AChE activity have shown above 75% inhibitions at 1 mg/mL, but decreased to <25% for most extracts at 0.25 mg/mL [73].

The BSG fractions and extracts tested for anti-AChE and BChE activities showed high and low inhibitory potential and corresponded to high or low contents of TPC and SQP (Table 3). This suggested that the phenolic compounds are possible effective natural inhibitors against AChE and BChE activities. Hence, the individual polyphenol and their blends were investigated for the enzyme inhibition studies.

Table 4 shows the AChE and BChE inhibitory potential (%) of individual phenolic compounds prepared at a specific concentration along with three blends that replicate their concentrations in BSG fractions. The activity of the various standard polyphenols at 1 mg/mL concentration was in the order: Caffeic acid > ferulic acid > *p*-coumaric acid, catechin, protocatechuic acid > 4-HBA for AChE inhibition, whereas for BChE the order of activity were caffeic acid > catechin > ferulic acid > *p*-coumaric acid > 4-HBA > protocatechuic acid. All the tested polyphenols at a 10-fold lower concentration presented an insignificant inhibition activity of <5% for AChE and <15% for BChE with some polyphenols expressing no inhibition at all. In general, the individual polyphenol showed a stronger inhibition against BChE than AChE at 1 mg/mL. Caffeic acid showed the most potent inhibitory activity with 52.3 ± 0.75% at 1 mg/mL against AChE and 25.5 ± 0.30% against BChE activity. The prepared polyphenol blends presented insignificant inhibition against both AChE and BChE activities at 1 mg/mL and lower inhibitions compared to their actual counterparts.

The composition of blends mimicked only the quantified individual polyphenols in the BSG FP EtOAc, BP DE, and BP EtOAc fractions, whereas these fractions could contain other unidentified compounds, i.e., peptides, amino acids, lipids. The difference in composition together with the quantified ferulic acid dimers and trimers in the fractions may explain the lower inhibitory potential of the BP DE and BP EtOAc blends against both AChE and BChE activities. The FP EtOAc fraction presented very low quantifiable polyphenols with catechin being the most abundant and representing 68% of the total quantified polyphenols. The FP EtOAc blend contained the same % of catechin but at higher content when tested against AChE and BChE activity. Both the fraction and blends showed similar inhibition for BChE activity, whereas low or no inhibition detected against AChE activity. Additionally, an explanation of the higher inhibitory potential of the fractions vs blends would be that the identified and unidentified compounds might present a synergistic effect in the fractions compared to blends, thus increasing their potency towards the inhibition of cholinesterases’ activities. 

### 3.5. Pearson Correlation, Multiple Regression Model of Variables and PCA

In order to understand whether there is an association between the above data sets specifically among pairs of variables, i.e., enzymatic assays AChE vs BChE, polyphenols quantification methods TPC vs SQP, or within sets of variables in particular FA versus *p*-CA, or FA versus AChE etc., a number of statistical tests have been performed.

Correlation tests have been performed to identify any relationships between variables (AChE, BChE, TPC, SQP, and individually quantified polyphenols) either in the BSG FP and BP extracts, or individual polyphenols and their blends. Depending on the independent variables used, the correlation values may increase or decrease. For example, the FP, BP extracts, individual standards and blends were tested for both AChE and BChE, whereas TPC and SQP were analyzed without the individual standard as no data was available. The correlation between the individual phenolic standards was analyzed only in the BP extracts.

AChE and BChE enzymatic assays presented a significant (*p* < 0.01) correlation of 0.687 (*n* = 84) determined by FP and BP extracts, polyphenol standards and blends. The quantification methods of polyphenols content in BSG extracts, TPC and SQP, presented a significant (*p* < 0.01) correlation of 0.974 (*n* = 48) determined by FP, BP extracts and blends. Furthermore, significant (*p* < 0.01) correlation has been observed in BP extracts (*n* = 21) between individual polyphenols, i.e., FA and *p*-CA (0.958), 4-HBA (0.994) and CafA (0.887), respectively. Moreover, the FA dimer, DeCa-DiFA presented significant correlation (*p* < 0.01) with the enzymatic assays AChE and BChE of 0.754 and 0.896, respectively (*n* = 21). Other related correlations are shown in (Table S2). The multiple correlation coefficient R indicated a very high correlation of 0.842 and 0.984 between the response variables, AChE and BChE, and the explanatory variables (TPC, FA, CafA, 4HBA, ProA, *p*-CA, Cat, DeCa-DiFA, TriFA), with the BP extracts. Further, the coefficient of determination (*R^2^*) indicates that the model fits the data reasonably well; where 70.9% (AChE) and 97.6% (BChE) of the variation could be explained by the fitted model. The adjusted R^2^ value of the dependent variable AChE considerably reduced the estimated proportion to 0.471 and slightly to 0.941 for BChE, respectively. A regression model has also been presented using FA and *p*-CA (most abundant polyphenols) as variables to explain the anti-AChE and BChE activity (Table S3).

**Figure 2:**
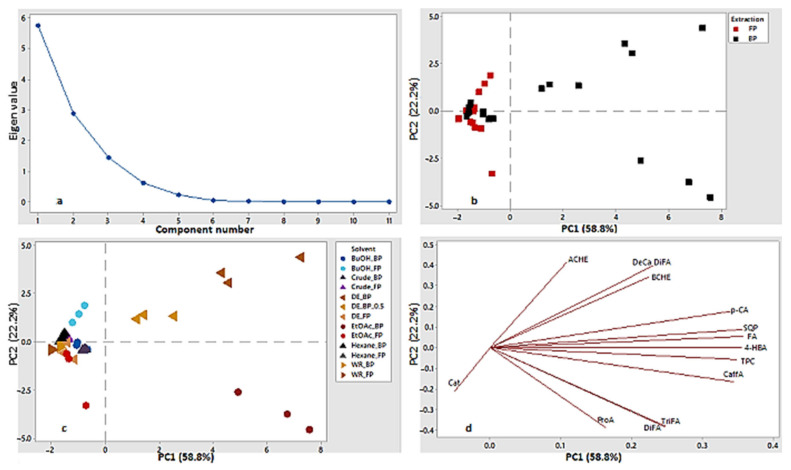
(**a**) Scree plot of BSG FP and BP extracts and fractions; (**b**) Score plot for the first two components (PC) separated by the type of extractions FP and BP; (**c**) score plot for the first two components separated by the type of organic solvent used for fractionation of the FP and BP extracts; (**d**) loading plot of the first two components.

Principle component analysis (PCA) was performed on standardized datasets to explore a potential differentiation among BSG FP and BP extractions and their follow-up fractions (Figure 2) based on individual polyphenol content (i.e., FA, *p*-CA, CafA etc.), polyphenol quantification methods (TPC, SQP) and enzymatic assays (AChE and BChE).

PC1 retained about 59% of data variation, while PC2 explained an extra 22% of overall variability leading to a total cumulative variation of 81%. Two score plots for PC1 and PC2 are presented in (Figure 2), where the variables were separated according to the type of extraction, FP and BP (Figure 2b), and further partitioning of the extracts by organic solvents, Hex, DE, EtOAc, BuOH, and WR, respectively (Figure 2c). In (Figure 2b), it can be observed the formation of a cluster close to the origin of the plot by both FP and BP fractions, and part separation of several BP fractions, in the upper and lower right-hand side of the plot. In (Figure 2c), the part separation is represented by the EtOAc BP fractions in the lower right-hand side, and DE BP fractions in the upper side. These two BP fractions seemed to have a stronger impact on the model as they are the furthest away from the plot’s origin.

The loading plot (Figure 2d) shows the relations between the analyzed variables including quantified phenolic acids, quantification methods and enzymatic assays, explained in combination with the eigen values (Table S4). Three sets of associations between variables were observed in the loading plot (Figure 2d). PC1 positively differentiated the BSG FP and BP fractions according to the contents of FA, *p*-CA, CafA, 4-HBA, and the polyphenol quantifications methods, i.e., TPC and SQP. This positive association was an expected result as a strong and significant Pearson correlation was observed between these variables (Table S2). FA and *p*-CA were the most abundant polyphenols in the BSG extracts and fractions, and with CafA and 4-HBA brought a higher contribution to TPC and SQP quantification methods compared to catechin. PC2 differentiated the BSG FP and BP fractions according to the contents of ProA, DeCa-DiFA, DiFA, TriFA, and the enzymatic assays AChE and BChE. The positive association between DeCa-DiFA and the enzymatic assays, AChE and BChE was an expected result too as among the quantified polyphenols, DeCa-DiFA presented a strong and significant Pearson correlation with both enzymatic assays compared to DiFA, TriFA, and ProA. Moreover, DeCa-DiFA was present only in the DE BP fraction, which presented the highest inhibition among the analysed fractions for both AChE and BChE activities. DiFA and TriFA were present only in EtOAc BP fraction, which presented a 4- and 2-fold lower inhibitions for AChE and BChE activities, respectively.

The statistical analysis showed significant correlations and strong associations between the analyzed variables of BSG fractions. A clear differentiation between BSG BP polyphenol-rich fractions and FP fractions was observed based on the performed statistical analysis with the most abundant polyphenols (i.e., FA, *p*-CA) being associated with the polyphenol quantification methods, and the decarboxylated FA dimer of BSG BP DE fraction associated with the anti-AChE and BChE activities. Szwajgier et al., 2012 have associated phenolic compounds from malt as potential cholinesterase inhibitors due to their similar structure to the well-known anti-cholinesterase, in terms of molecular weight, phenol rings and hydrophobic moieties. The highest anti-ChE activities was exhibited by *p*-coumaric acid at 0.38 mM/L, whereas the second best ferulic acid presented a 120-fold lower inhibition at 1 mM/L. In the same study, sinapic and 4-hydroxybenzoic acid (0.03 and 0.01 mM/L) presented similar inhibitions to ferulic acid [74]. In a subsequent study by Szwajgier et al., 2013, ferulic acid and *p*-coumaric acid showed similar level of anti-AChE and anti-BChE activities at 0.2 mM, whereas caffeic acid showed slightly higher inhibitory potential against AChE and lower for BChE [75]. The work of Shahwar et al., 2010 have showed ferulic acid to exhibit AChE inhibitions of 12.38 to 42.65% at varying concentrations (50 to 250 μg/mL) and was found to be strongly dose dependent and with no significant change in inhibition at concentrations above 250 µg/mL [76]. As it can be seen in (Table 4), FA and *p*-CA presented similar levels of inhibitions towards both enzymes at 0.1 and 1 mg/mL, respectively. Contrary to Szwajgier et al., 2012 study, Ouattara et al. 2013 showed no inhibitory effect on AChE activity by *p*-coumaric acid [71]. Interestingly, caffeic acid at 1 mg/mL showed no inhibition against AChE or BChE in the study by Orhan et al., 2007 [77], whereas in this work caffeic acid showed the highest activities against both cholinesterases. Caffeic acid has been previously shown to present a higher antioxidant activity than other hydroxycinnamic acids, i.e., FA, *p*-CA [78], thus may explain the higher inhibitory potential towards the cholinesterases. Vladimir et al., 2014 have also examined individual hydroxycinnamic acids, and they presented a stronger AChE inhibition than the hydroxycinnamic acid rich plant extracts. For example, ferulic acid showed a ~50%, ~75%, and ~87% AChE inhibition, and caffeic acid, like in our present study, showed a ~30%, ~85, and ~90%, at 0.25, 0.5 and 1 mg/mL concentrations, respectively [73].

The insignificant anti-AChE and BChE activities of prepared blends would need to be investigated further as the interactions among phenolic compounds could be synergistic or antagonistic, and those studies are sparse and lacking. As an example, the interaction between *p*-coumaric and ferulic acid in respect to antioxidant capacity is additive, but when caffeic acid is present, the type of interaction changes to antagonistic [79].

Galantamine, an alkaloid isolated from *Galanthus Woronowii* currently used in AD treatment, is a centrally acting reversible and competitive inhibitor of cholinesterases. Galantamine has shown a 53-fold greater inhibitory activity for AChE than BChE (IC_50_ values ranging from 0.1 to 5.3μg/mL) [80]. High anti-AChE potency of alkaloids is attributed to the binding of its quaternary nitrogen to an aspartate residue at AChE peripheral anionic site [81], or the ability to build hydrogen bonds with Tyr130 [82], and also due to a hydroxyl group at the alkaloid C-2 position [83]. On the other hand, polyphenols and terpenes bind to the peripheral anionic site of AChE acting as non-competitive inhibitors [84]. Santos et al., 2018 have reviewed several papers related to anti-ChE activities in which a total of 54 plants species with 36 isolated bioactive compounds were investigated; the authors revealed that alkaloids and coumarins presented a higher potency (IC_50_ <20μg/mL) than galantamine (IC_50_ of 5 μM), whereas flavonoids and phenolic acids presented low potency (IC_50_ 50–1000μM) [84]. Furthermore, phenolic compounds with close molecular weights (254.24–354.40 Da) have showed that the enzyme-inhibitory activity decreased by the presence 3-hydroxyl group, whereas other hydroxyl groups, their position and number, played a minor role in this context [85].

Roasting temperatures (>150 °C) have shown to increase the levels of catechin and proanthocyanidin hexamers and heptamers in cacao processing, which further improved the inhibitory potential of extracts against enzyme activity [86]. High temperature roasting (<232 °C) is also applied in barley malt to produce stout beer resulting in BSG dark residues. Extracts obtained from BSG dark may possess increased levels of homogenous and heterogenous oligomers of phenolic compounds, which may attribute to improve their potential as enzyme inhibitors.

Another important observation in this study was that the EtOH at concentrations of <10% in the final assay mixture interfered with the enzymes activity and resulted inhibitions of up to 30% for AChE and less than 4% for BChE activity, respectively. As the tested extracts had been reconstituted in 0 to 50% ethanol (0 to 5% in final assay mixture), the observed EtOH inhibition was subtracted from the final calculations. Several authors have presented numerous polar and non-polar organic solvents that might decrease or enhance the cholinesterase activities [87,88], but this was not the purpose of the current study.

With regard to AD management, the AChE and BChE inhibition are still attractive targets owing not only to the cholinergic hypothesis but as well to several functions in pathogenesis and development of AD [89]. Approximatively 95% of the cholinesterase activity is due to AChE in normal human brain, whereas its level decreases to 10–15% in the brain of a person with AD, and interestingly BChE activity increases to 120% [89].

The molecular mechanism of interaction of alkaloids is similar to the currently used drugs for this purpose, i.e., huperzine, galantamine, thus the high anti-AChE potency [84]. Phenolic compounds are currently considered as a noticeable agents of reduced risk and management of AD due to their antioxidant, anti-inflammatory and anti-cancer capacities, low toxicity and abundant sustainable natural sources [15,17,18].

## 4. Conclusions

BSG represent a clear opportunity to be exploited as a potential source of bioactive compounds if processed in the right way, and further its corresponding polyphenolic extracts be accepted and utilized in health and food processing.

In the current study, BSG extracts and their sub-fractions along with commercially pure phenolic compounds and blends of identified BSG polyphenols were tested for their potential to inhibit AChE and BChE activities in vitro. Saponification with NaOH (bound phenolic extract) presented the highest polyphenol content per gram of BSG in DE and EtOAc fractions as revealed by TPC (FC reagent) and SQP (UPLC-MS/MS). Ferulic- and *p*-coumaric acids were the most abundant polyphenols, with the highest levels in the DE and EtOAc bound phenolic fractions, whereas catechin was the most abundant in the same solvent fractions but as free phenolics. These results indicate the necessity of using alkali hydrolysis followed by liquid–liquid partitioning with DE and EtOAc to obtain high polyphenol yields.

The in vitro enzymatic assays revealed that not only polyphenol rich fractions (BP DE and BP EtOAc) significantly inhibited AChE and BChE activities, but low polyphenolic-containing fractions (FP BuOH fraction) also had significant impact. Among the individually tested polyphenols, caffeic acid presented the highest inhibitory potential; however, its content in BSG is low. There seems to be a synergistic interaction between polyphenols and other co-extracted compounds in the BSG BP (DE and EtOAc) fractions, whereas little or no synergistic effect between the selected polyphenols in the blend for cholinesterase inhibition. The PCA analysis showed a strong inhibitory influence of the presence of a single compound DeCa-DiFA in DE fractions. Significant correlations (*p* < 0.01) have been observed between the enzymatic assays AChE and BChE, as well as between analysis methods TPC and SQP, normally used in concomitance in this type of research investigation and between the individual polyphenols (FA and *p*-CA). The inhibitory effect of BSG extracts and fractions, including their individual polyphenols, on AChE and BChE activity would require further studies such as an additional separation of compounds to identify the most potent compound(s).

## Figures and Tables

**Table 1:** Extraction yield (means ± SD) of free phenolics (FP) using 60% acetone and bound phenolic (BP) using 0.75% NaOH expressed in milligram per gram of BSG dry weight (mg/g BSG dw).

Samples	Extraction Yield (mg/g BSG)		TPC (mg GAE/g BSG)		SQP (mg/g BSG)	
	FP	BP	FP	BP	FP	BP
Hex F.	19.4 ± 16.8 ^a^	12.2 ± 7.1 ^b^	0.09 ± 0.09 ^a^	0.02 ± 0.0 ^b^	n.d. ^b^	<0.01 ^c^
DE F.	25.8 ± 14.2 ^a^	8.3 ± 1.2 ^b^	0.23 ± 0.09 ^a^	0.67 ± 0.02 ^b^	<0.01 ^a^	0.8 ± 0.0 ^a b^
EtOAc F.	6.6 ± 4.0 ^a^	34.6 ± 6.9 ^a^	0.12 ± 0.07 ^a^	3.5 ± 0.5 ^a^	<0.01 ^a^	2.9 ± 0.3 ^a^
BuOH F.	6.2 ± 4.4 ^a^	23.8 ± 8.3 ^a b^	0.09 ± 0.004 ^a^	1.0 ± 0.4 ^a b^	<0.01 ^a b^	0.07 ± 0.03 ^b c^
WR F.	22.2 ± 7.8 ^a^	345 ± 162.5 ^a^	0.11 ± 0.02 ^a^	6.2 ± 2.8 ^a^	n.d. ^b^	0.03 ± 0.04 ^c^
Total	80.2 ± 3.4	424.2 ± 179.9	0.64 ± 0.07	11.3 ± 3.6	0.013 ± 0.02	3.80 ± 0.2
Crude (Control)	94.9 ± 9.2	n.t.	1.7 ± 0.2	n.t.	<0.02	n.t.

Total represents the sum of each solvent fraction (F.) in the column. Fractions generated by Hex—hexane, DE—diethyl ether, EtOAc—ethyl acetate, BuOH—butanol and WR (water residue); “n.t.” means not tested, “n.d.” means not detected. The corresponding polyphenols content in FP and BP samples represented by Total Phenolic Content (TPC) by Folin–Ciocalteu in mg of gallic acid equivalent per gram of BSG (mg GAE/g BSG), and sum of quantified polyphenols (SQP) by UPLC-MS/MS in mg/g BSG. Values in the same column for each type of extracted phenolics (FP and BP) with each solvent fraction (Hex, DE, EtOAc, BuOH, WR) bearing different letters (a, b, c) are significantly different (*p* < 0.05) from each other.

**Table 2:** Individual polyphenols quantified by UPLC-MS/MS in microgram/milligram (μg/mg) BSG extract.

Samples (µg/mg)	FA		*p*-CA		Cat		CafA		4-HBA		ProA		DeCa-DiFA		DiFA		TriFA		Total	
	FP	BP	FP	BP	FP	BP	FP	BP	FP	BP	FP	BP	FP	BP	FP	BP	FP	BP	FP	BP
Hex F.	n.d.	0.04 ± 0.07 ^b^	n.d.	0.05 ± 0.06 ^c^	n.d.	n.d.	n.d.	n.d.	n.d.	n.d.	n.d.	n.d.	n.d.	0.08 ± 0.08 ^b^	n.d.	n.d.	n.d.	n.d.	n.d.	0.15 ± 0.2 ^c^
DE F.	0.053 ± 0.03 ^a^	41.5 ± 15.3 ^a^	0.03 ± 0.02 ^a^	25.9 ± 6.3 ^a^	0.33 ± 0.10 ^a^	n.d.	0.02 ± 0.01 ^a^	0.50 ± 0.2 ^a b^	0.05 ± 0.05 ^a^	0.30 ± 0.1 ^a b^	0.06 ± 0.03 ^ab^	0.04 ± 0.00 ^a b^	n.d.	30.62 ± 0.8 ^a^	n.d.	0.04±0.01 ^b^	n.d.	n.d.	0.5 ± 0.3 ^a^	99.0 ± 21.2 ^a^
EtOAc F.	0.054 ± 0.03 ^a^	40.6 ± 7.7 ^a^	n.d.	16.1 ± 1.9 ^a b^	0.88 ± 0.67 ^a^	n.d.	0.03 ± 0.02 ^a^	1.04 ± 0.3 ^a^	0.04 ± 0.03 ^a^	0.33 ± 0.05 ^a^	0.21 ± 0.04 ^a^	0.24 ± 0.06 ^a^	n.d.	0.95 ± 0.6 ^a b^	n.d.	12.91±2.4 ^a^	n.d.	12.76 ± 2.3 ^a^	1.0 ± 0.9 ^a^	84.9 ± 14.6 ^a b^
BuOH F.	n.d.	1.2 ± 1.0 ^a b^	n.d.	0.44 ± 0.4 ^b^	0.06 ± 0.06 ^a^	n.d.	n.d.	0.03 ± 0.02 ^b c^	n.d.	0.02 ± 0.01 ^b^	0.03 ± 0.03 ^a b^	0.03 ± 0.01 ^b^	n.d.	0.08 ± 0.04 ^b^	n.d.	0.92±0.6 ^a b^	n.d.	0.70 ± 0.7 ^a b^	0.07 ± 0.1 ^a^	3.3 ± 2.8 ^b c^
WR F.	n.d.	n.d.	n.d.	n.d.	n.d.	n.d.	n.d.	n.d.	n.d.	n.d.	n.d.	n.d.	n.d.	n.d.	n.d.	0.09 * ^b^	n.d.	0.11 ^* b^	n.d.	0.1 ± 0.1 ^c^
Crude Ctrl	n.d.	5.33 *	n.d.	2.28 *	0.15 *	n.d.	n.d.	0.08 *	0.01 *	0.03 *	0.02*	0.02*	n.d.	0.58 *	*-*	1.09 *	n.d.	0.78 *	0.19 *	10.2 *

Individual phenolic compounds represented by ferulic acid (FA), p-coumaric acid (p-CA), catechin (Cat), caffeic acid (CafA), 4-hydroxybenzoic acid (4-HBA), protocatechuic acid (ProA), decarboxylated diferulic acid (DeCa-DiFA), diferulic acid (DiFA) and triferulic acid (TriFA), in BSG Free Phenolic (FP) and Bound Phenolic (BP) extracts and their organic solvent fractions (F.), hexane (Hex), diethyl ether (DE), ethyl acetate (EtOAc), butanol (BuOH), water residue (WR), and Crude control (Ctrl). “n.d.”—not detected, “*”—identified in one of the extracts. The values reported for each individual polyphenols and Total in FP and BP extracts with their solvent fractions bearing different letters (a, b, c) are significantly different (*p* < 0.05) from each other.

**Table 3:** Total phenolic content (TPC), sum of quantified polyphenols (SQP) of free phenolic (FP) and bound phenolic (BP) extracts and their anticholinesterase activities in different solvent fractions tested at 1 mg/mL.

Samples 1 mg/mL		TPC μgGAE/mg Extract	SQP μg/mg Extract	AChE %Inhibition	BChE %Inhibition
FP	Hex F.	4.1 ± 0.6 ^e^	n.d.	11.7 ± 1.3 ^b^	17.5 ± 1.8 ^c d^
	DE F.	9.8 ± 1.9 ^e^	0.5 ± 0.3 ^b^	10.7 ± 3.6 ^b^	16.4 ± 3.1 ^c d^
	EtOAc F.	20.3 ± 3.4 ^d e^	1.0 ± 1 ^b^	8.7 ± 0.6 ^b^	15.7 ± 2.9 ^c d^
	BuOH F.	11.4 ± 2.5 ^e^	0.07 ± 0.1 ^b^	34.9 ± 6.4 ^a^	40.5 ± 11.2^b^
	WR F.	5.1 ± 1.1 ^e^	-	12.8 ± 0.7 ^b^	-
	Crude Ctrl	17.3 ± 0.7 ^d e^	0.19 * ^b^	20.8 ± 2.2 ^b^	17.2 ± 1.2 ^c d^
BP	Hex F.	1.8 ± 0.4 ^e^	0.15 ± 0.2 ^b^	13.8 ± 3.5 ^b^	25.1 ± 1.5 ^c^
	DE F.	82.9 ± 13.2 ^b^	99.0 ± 21.2 ^a^	37.9 ± 10.4 ^a^	53.6 ± 7.7 ^a^
	EtOAc F.	102.3 ± 14.1 ^a^	84.9 ± 14.6 ^a^	10.3 ± 2.9 ^b^	25.3 ± 3.3 ^c^
	BuOH F.	40.7 ± 1.6 ^c^	3.3 ± 2.8 ^b^	14.3 ± 2.9 ^b^	16.9 ± 3.1 ^c d^
	WR F.	18.0 ± 0.9 ^d e^	0.1 ± 0.1 ^b^	11.6 ± 1.3 ^b^	9.4 ± 3.8 ^d e^
	Crude Ctrl	31.7 ± 0.8 ^c d^	10.2 * ^b^	10.2 ± 1.4 ^b^	11.4 ± 0.4 ^d e^

Hex—hexane, DE—diethyl ether, EtOAc—ethyl acetate, BuOH—butanol, WR—water residue, F- fraction, Crude Ctrl—crude control, n.d.—not detected. TPC by Folin–Ciocalteu; SQP by UPLC-MS/MS; Acetyl -, Butyrylcholinesterase (AChE, BChE) inhibition activity expressed as % inhibition and compared to galantamine at IC_50_ (50% inhibition by 3.4 ± 0.23μg/mL for AChE and 11.9 ± 1.67 μg/mL for BChE). The data with an * in the SQP column is given as a single result. The values reported on the column for each TPC, SQP, AChE and BChE in FP and BP crude extracts with their solvent fractions bearing different letters (a–e) are significantly different (*p* < 0.05) from each other.

**Table 4:** The potential of six individual polyphenols at 0.1 and 1 mg/mL towards the inhibition (%) of acetylcholinesterase (AChE) and butyrylcholinesterase (BChE) activities.

Standards	AChE % Inhibition		BChE % Inhibition	
	0.1 mg/mL	1 mg/mL	0.1 mg/mL	1 mg/mL
Ferulic A.	1.0 ± 0.9 ^b^	15.4 ± 0.1 ^a b^	14.6 ± 1 ^a b^	27.2 ± 0.9 ^a b^
***p***-Coumaric A.	5.2 ± 0.4 ^a^	14.4 ± 0.5 ^b c^	6.4 ± 0.6 ^b^	22.1 ± 1.3 ^b c^
Catechin	3.8 ± 1.1 ^ab^	14.9 ± 0.2 ^a b^	12.2 ± 0.6 ^a b^	31.6 ± 0.4 ^a b^
4-Hydroxybenzoic A.	1.0 ± 0.2 ^b^	5.2 ± 0.9 ^c^	n.d.	11.9 ± 0.6 ^b c^
Caffeic A.	3.3 ± 0.4 ^a b^	25.5 ± 0.2 ^a^	15.4 ± 1.3^a^	52.3 ± 0.8 ^a^
Protocatechuic A.	n.d.	13.8 ± 0.7 ^b c^	n.d.	7.6 ± 2.4 ^c^
**Blends**	**TPC** **μgGAE/mg**	**SQP** **μg/mg**	**AChE** **%Inhibition**	**BChE** **%Inhibition**
FP EtOAc1	260.6 ± 11.9 ^a b^	1000	n.d.	16.7 ± 1.5 ^a^
BP DE1	243.8 ± 1.4 ^b^	1000	11.1 ± 0.6 ^a^	9.9 ± 0.2 ^b^
BP EtOAc3	267.4 ± 8.4 ^a^	1000	8.3 ± 0.1 ^a^	11.2 ± 1.1 ^a b^

Three blends (FP EtOAc1, BP DE1, BP EtOAc3), that mimic the polyphenol content in the BSG fractions with the highest Total phenolic content (TPC) and sum of quantified polyphenols (SQP) were tested as well. The values reported for each AChE and BChE at specific concentrations with their individual polyphenols bearing different letters (a, b, c) are significantly different (*p* < 0.05) from each other. n.d. = not detected.

## Data Availability

Not applicable.

## Supplementary Materials

The following are available online at https://www.mdpi.com/article/10.3390/foods10050930/s1, Table S1: Blends of individual polyphenols at 1 mg/mL mimicking their abundance in BSG fractions; Table S2: Correlation coefficients among analyzed variables of BSG BP fractions; Table S3: Summary of multiple regression model of AChE and BChE; Table S4: The first four factor loadings for illustrating the interpretation of (Figure 2).
