# Supplementary material for: Anticholinesterase Activities of Different Solvent Extracts of Brewer’s Spent Grain
Source: Foods. 2021 Apr 23;10(5):930. doi: 10.3390/foods10050930 (PMC8145039; doi:10.3390/foods10050930)
Supplement: Supplementary file 1 [file foods-10-00930-s001.zip › foods-1173862-supplementary.pdf]

foods MDPI

Article

Anticholinesterase Activities of Different Solvent Extracts of Brewer's Spent Grain

Rares I. Birsan [1,2], Peter Wilde [2], Keith W. Waldron [3] and Dilip K. Rai [1,*]

[1] Department of Food BioSciences, Teagasc Food Research Centre Ashtown, D15KN3K Dublin, Ireland; Rares.Birsan@teagasc.ie
[2] Food Innovation and Health Programme, Quadram Institute Bioscience, Norwich Research Park, Colney NR4 7UQ, UK; Pete.Wilde@quadram.ac.uk
[3] Anglia Science Writing Ltd., Wramplingham NR18 0RU, UK; keithwwaldron@outlook.com
* Correspondence: dilip.rai@teagasc.ie; Tel.: +353-01-805-9500

Supplementary Materials:

Table S1. Blends of individual polyphenols at 1 mg/mL mimicking their abundance in BSG fractions.

Sample	Total (µg/mL)	FA	*p*-CA	Cat	CafA	4-HBA	ProA
Blend FP EtOAc 1	1000	36.6	3.7	683.7	23.5	35.5	217
Blend BP DE 1	1000	633.4	354.5	-	7.1	4.5	0.5
Blend BP EtOAc 3	1000	703.2	267.6	-	19.9	5	4.3

FP—free phenolic extract, BP—bound phenolic extract followed by the fraction replicate number; µg/mL—microgram per milliliter; FA—ferulic acid, *p*-CA—coumaric acids, Cat—catechin, CafA—caffeic acid, 4-HBA—hydroxybenzoic acid, ProA—protocatechuic acid.

Table S2. Correlation coefficients among analyzed variables of BSG BP fractions.

Pearson Correl.	AChE	BChE	TPC	SQP	FA	p-CA	Cat	CafA	4-HBA	ProA	DeCa-DiFA	DiFA	TriFA
AChE	1												
BChE	0.687**	1											
TPC	0.375	0.511*	1										
SQP	0.543*	0.787**	0.904**	1									
FA	0.502*	0.736**	0.916**	0.990**	1								
p-CA	0.645**	0.869**	0.826**	0.980**	0.958**	1							
Cat	−0.036	−0.013	−0.293	−0.194	−0.189	−0.187	1						
CafA	0.163	0.428	0.922**	0.856**	0.887**	0.739**	−0.163	1					
4HBA	0.461*	0.715**	0.927**	0.985**	0.994**	0.941**	−0.190	0.907**	1				
ProA	−0.113	0.094	0.801**	0.610**	0.656**	0.439*	−0.139	0.922**	0.700**	1			
DeCa-DiFA	0.754**	0.896**	0.432	0.703**	0.621**	0.817**	−0.135	0.256	0.585**	−0.095	1		
DiFA	−0.229	−0.052	0.702**	0.481*	0.538*	0.298	−0.107	0.854**	0.585**	0.983**	−0.253	1	
TriFA	−0.227	−0.040	0.700**	0.488*	0.545*	0.306	−0.102	0.858**	0.595**	0.985**	−0.245	0.999**	1

Correlation is significant at the 0.01** level and at the 0.05* level (2-tailed).

Table S3. Summary of multiple regression model of AChE and BChE.

Model	*R*	*R* Square	Adjusted R Square	Std. Error of the Estimate
AChE	0.842[a]	0.709	0.471	7.55749
AChE	0.763[b]	0.582	0.536	7.08868
BChE	0.984[a]	0.967	0.941	3.74240
BChE	0.933[b]	0.871	0.856	5.82498

[a]Predictors: (Constant), FA, *p*-CA, Cat, CafA, 4HBA, ProA, DeCA-DiFA, TriFA, TPC; [b]Predictors: (Constant), FA, *p*-CA.

Table S4. The first four factor loadings for illustrating the interpretation of figure 2. Note: Numbers in bold represent the factor loading higher than 0.30.

Variable	PC1	PC2	PC3	PC4
FA	0.357	0.053	0.020	0.113
p-CA	0.340	0.176	−0.041	0.205
Cat	−0.049	−0.209	−0.765	0.004
CafA	0.343	−0.167	0.096	−0.028
4−HBA	0.356	−0.001	−0.068	0.111
ProA	0.163	−0.388	−0.488	−0.125
DeCa−DiFA	0.229	0.395	−0.164	0.362
DiFA	0.247	−0.383	0.226	−0.223
TriFA	0.248	−0.383	0.222	−0.224
ACHE	0.108	0.410	−0.087	−0.737
BCHE	0.223	0.339	−0.154	−0.344
TPC	0.347	−0.057	0.034	0.035
SQP	0.356	0.087	−0.004	0.148
Eigenvalue	7.600	2.882	1.4534	0.636
Explained variance (%)	58.5	22.2	11.2	4.9
Cumulative variance (%)	58.5	80.7	91.9	96.8
